# Tubulovillous Adenoma in the Bladder in a Dual Pancreas-Kidney Transplant Patient

**DOI:** 10.1089/cren.2016.0139

**Published:** 2017-02-01

**Authors:** Taylor Remondini, Stephan Van Zyl, Tarek A. Bismar, Serdar Yilmaz, M. Eric Hyndman

**Affiliations:** ^1^ Undergraduate Medicine, University of Calgary, Calgary, Canada.; ^2^ Department of Urology, Southern Alberta Institute of Urology, Calgary, Canada.; ^3^ Department of Pathology and Laboratory Medicine, University of Calgary, Calgary, Canada.; ^4^ Department of Transplant Surgery, University of Calgary, Calgary, Canada.

**Keywords:** bladder cancer, pancreas-kidney transplant, tubulovillous adenoma, cystoscopy, transurethral resection

## Abstract

***Background:*** A rare report of a tubulovillous adenoma arising in the setting of a dual pancreas-kidney transplant patient.

***Case Presentation:*** This adenoma was discovered in a 60-year-old male with a dual pancreas-kidney transplant that presented with urinary retention and gross hematuria. Management of this patient required both transurethral resection of the tumor as well as a laparotomy after recurrence. Follow-up with cystoscopy has shown no further recurrence of the tumor.

***Conclusion:*** This case adds to the few cases documented of adenomas arising in bladders augmented with gastrointestinal tract tissue. The tumor may reflect growth from donor duodenal graft tissue, however, the metaplasia of urothelial tissue cannot be fully ruled out. Based on this case, our understanding of these rare tumors and their clinical course is deepened.

## Case Presentation

A 60-year-old male presented to the emergency department with urinary retention. After a catheter was placed in the patient's bladder gross hematuria was observed. The patient's medical history is significant for a dual pancreas and kidney transplant 16 years before this emergency department presentation. He received these transplants due to end-stage renal disease caused by type 1 diabetes mellitus. His graft function has been stable since transplant and he no longer requires insulin. He does, however, have retinopathy, nephropathy, and neuropathy complications associated with his Type 1 diabetes diagnosis. The patient has had a colonoscopy for unintended weight loss performed a year before this emergency department visit that revealed no visual abnormalities. Upon physical examination, his vital signs were found to be stable and there were no signs of infection. He did have suprapubic tenderness.

**FIG. 1.**
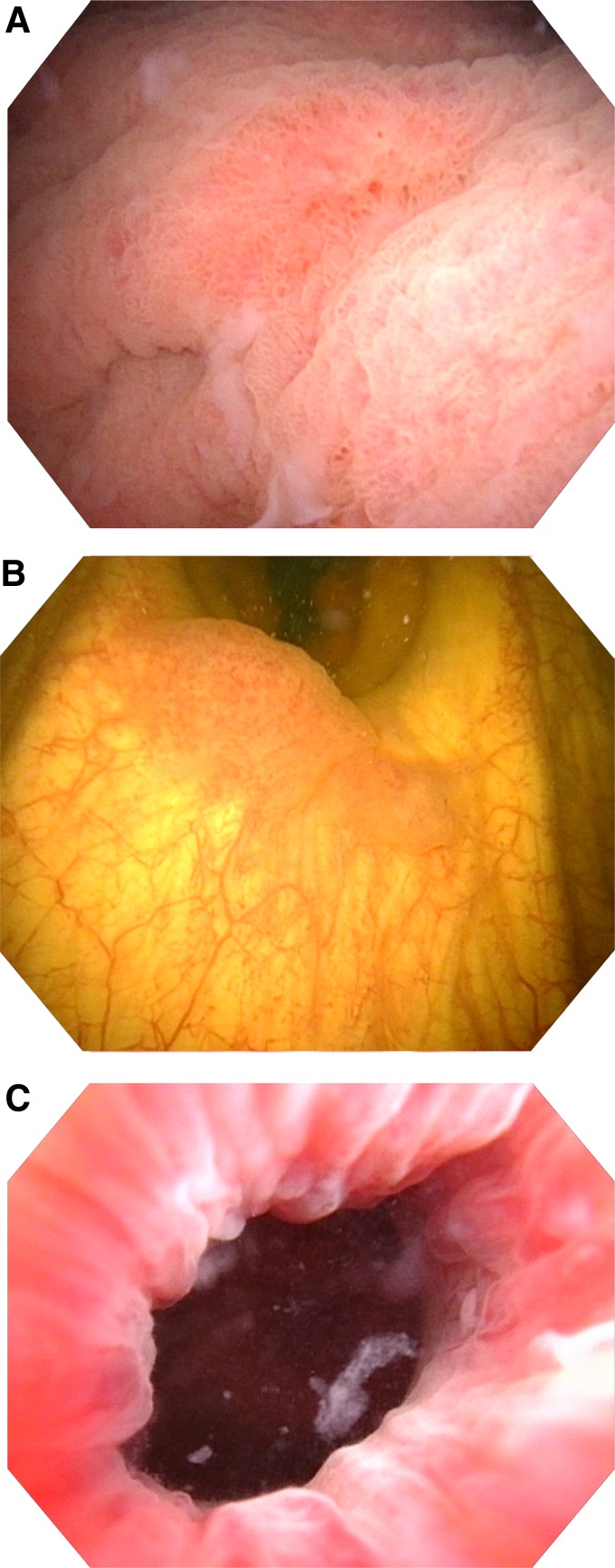
Cystoscopy diagnosis of the tumor. **(A)** Tumor site at the transplant ureteral orifice. **(B)** Retroflexed view of the tumor. **(C)** View of the bladder neck showing no visible malignancy.

The serum creatinine was 114 μmol/L with an estimated glomerular filtration rate (GFR) of 60 when this patient presented to the emergency department. No abnormalities were noted in the rest of the patient's blood work. The patient proceeded to undergo flexible cystoscopy to identify the source of the hematuria. A lesion that was highly suspicious for urothelial carcinoma was identified at the ureteral orifice (Fig. 1). It did appear that the lesion was coming from either the transplant ureter or transplant junction. The rest of the procedure was unremarkable apart from a false passage in the urethra likely from a past catheter insertion. A follow-up CT scan showed no hydronephrosis or urothelial thickening in the transplanted kidney. No mass lesion in the bladder could be identified, however, there was some mild thickening in the dome of the bladder, which was suspected to be inflammatory changes due to pancreatic duct dilatation.

Given the findings on cystoscopy, a transurethral resection of the bladder tumor was recommended for the patient. The patient was counseled regarding treatment options and informed consent was obtained for the procedure. The patient's antirejection medications were modified in anticipation of the upcoming surgery. After the patient had received appropriate antibiotics and a general anesthetic, a 26F resectoscope was introduced into the patient's urethra. The tumor was identified after entering the bladder. It had a relatively small stalk adjacent to bowel mucosa that was protruding into the bladder from the graft. The tumor was resected and sent to pathology for analysis. In addition, multiple cold-cup biopsies were taken from the resection site and around the bladder. The conduit was gently inspected to ensure that no perforation had occurred during the procedure. A 20F two-way Foley catheter was placed at the end of the procedure, and the patient was sent to recovery in good condition.

**FIG. 2.**
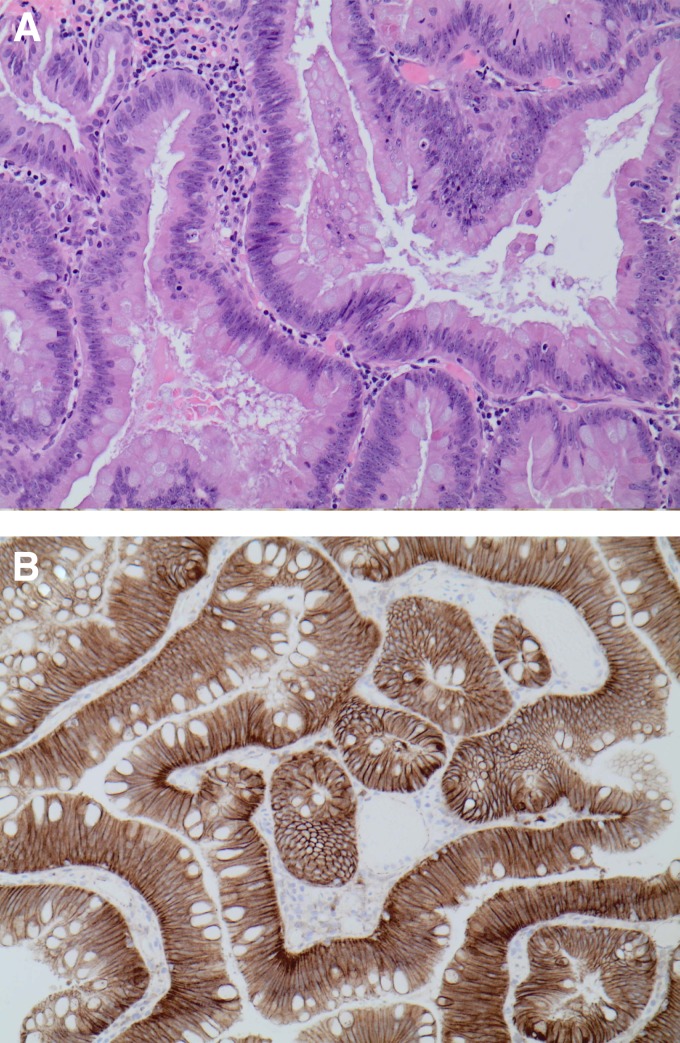
**(A)** High power view of the tubulovillous adenoma. It appears to be typical enteric or intestinal type adenoma with no high-grade dysplasia. **(B)** The lesion stains positive for β-catenin as would be expected for a tubulovillous adenoma. The lesion also stained positive for CDX-2.

Analysis of the pathological specimens following the procedure revealed that the resected lesion at the conduit site was not a urothelial carcinoma, but instead a tubulovillous adenoma (Fig. 2). This tumor is typical for an enteric-type adenoma likely arising from the duodenal graft from the donor instead of the bladder. There was no indication of high-grade dysplasia or invasive carcinoma in the polyps. The rest of the biopsies of the bladder did not reveal any malignant changes. Following the procedure, the patient's renal function remained stable with a creatinine of 105 μmol/L and an estimated GFR of 67 mL/minute.

**FIG. 3.**
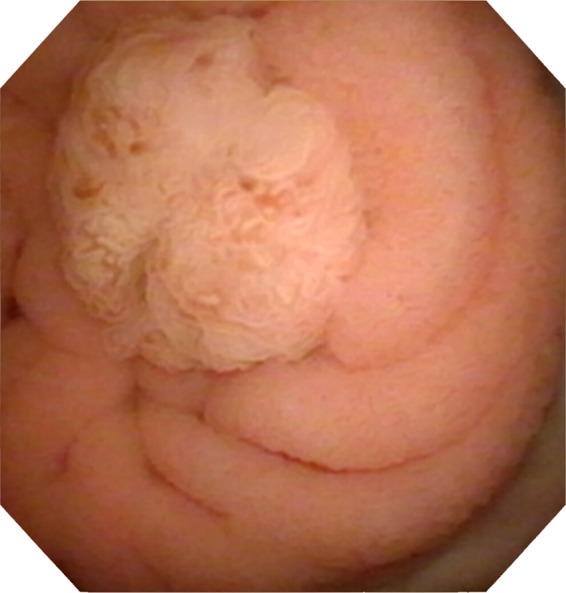
Follow-up cystoscopy showing recurrence of the tumor near the previous resection site.

The patient was followed up with cystoscopy 3 months after the operation to reevaluate the site for any signs of recurrence. At this time a small tumor was noted near the previous site of resection but farther up the conduit (Fig. 3). It is unknown whether this represents a new tumor or a recurrence of the previously resected tumor. The pathology for this resection revealed a tubular adenoma. Imaging with a CT scan and a chest X-ray did not reveal any disease outside of the conduit and bladder. A 7 mm tumor was noted in the conduit in keeping with the cystoscopy imaging.

**FIG. 4.**
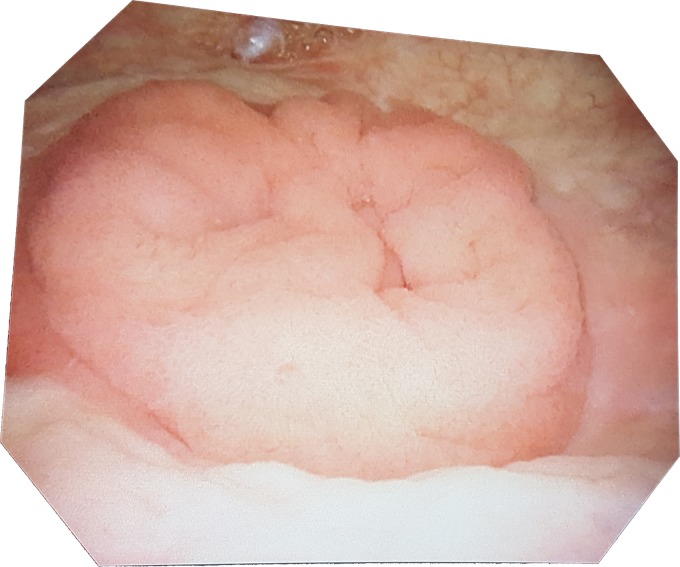
Follow-up cystoscopy showing no further recurrence of the tumor.

After considering these findings and discussing them with the patient as well as a transplant surgeon colleague, it was determined that the recommended treatment was a laparotomy and excision of this tumor in an open manner. An incision was made inferior to the urachus with cautery down through the fascia. The space of Retzius was developed and a catheter was placed in the surgical field allowing for the insufflation of the bladder. A cystoscope was then introduced allowing the visualization of the dome of the bladder. The bladder was then spatulated with care being given to preserve the pancreas conduit and the transplant ureter. The conduit junction was identified and inverted bluntly to allow direct visualization of the tumor. The tumor was then excised and the excision site was oversewn. Direct visualization did not reveal any other tumor tissue within the conduit. A frozen section of the tumor base revealed negative surgical margins during the surgery. These frozen pathology sections revealed a tubulovillous adenoma with low grade dysplasia. The incisions made were closed and the patient was sent to the recovery room in good condition with minimal blood loss. His postoperative stay in hospital was uneventful apart from some nausea and vomiting. The patient's most recent follow-up cystoscopy, however, shows no signs of regrowth (Fig. 4). No further biopsies were taken on this cystoscopy.

The patient is to be followed with serial cystoscopies every 6 months for the next 3 years to reevaluate the site for any signs of recurrence. If recurrence should occur, further surgical management options will be decided upon at that time. It is not believed that the rest of this patient's gastrointestinal tract is at significantly increased risk of tumor formation due to this finding as this tumor arose either donor and/or bladder tissue. Follow-up will be continued with the patient's transplant surgeon and nephrologist at his regularly scheduled appointments at the transplant clinic.

## Discussion

This case represents a unique and difficult to manage presentation of a tumor in a patient who received a dual kidney and pancreas transplant. Tubulovillous adenomas are a form of polyp and precursor to carcinoma that is generally found within the gastrointestinal tract. There have been few documented reports in scientific literature of this form of adenoma forming in bladders augmented with tissue from the gastrointestinal tract.^1^ In these cases, a cystoplasty was performed and tubulovillous adenoma was noted at the sites of anastomosed intestinal tissue. It was proposed in this case that malignant changes in the intestinal segments themselves could have resulted from a combination of chronic inflammation and *N*-nitrosamines produced by nitrate reduction by bacteria.

Another case of tubulovillous adenoma forming within a bladder was in the context of a gastrocystoplasty performed in a patient with neurogenic bladder symptoms.^2^ It was believed that this tubulovillous adenoma and focal adenocarcinoma transformation occurred within the bladder tumor as a result of tissue metaplasia. This conclusion was made given that the intestinal architecture remained intact. In addition, the presence of immunohistochemical marker CK7 in the tissue decreased the possibility of a colonic origin, however, it may be expressed in gastric adenocarcinoma. In keeping with this case, the tumor occurred at the site of the enterovesicular junction. Tissue metaplasia to form a tubulovillous adenoma within native bladder tissue has been noted in the context of inflammation from chronic cystitis previously.^3^

In our patient, it is believed based on the location of the tubulovillous adenoma that the tissue arose from the duodenal graft. Small bowel malignancies are uncommon in themselves making up 1%–2% of gastrointestinal malignancies.^4^ The duodenum itself accounts for 24.6%–33% of small bowel tumors. Small bowel cancer is most commonly histologically adenocarcinoma. A tubulovillous adenoma is in keeping with this, as it is a precursor to adenocarcinoma. Based on a literature search of PubMed and Medline it is believed that this is the first documented case of a tubulovillous adenoma arising from donor duodenal graft tissue in a dual kidney and pancreas transplant. The possibility of metaplasia of urothelial tissue near the orifice cannot be completely ruled out, however. Moving forward with this patient, it will be critically important to ensure that the patient is adequately monitored to ensure that any potential future changes within both the bladder and graft are identified and treated promptly. We are planning to continue with regular cystoscopies every 6 months to continue to follow this patient.

## Conclusion

The patient seen in this case presented with a unique and difficult to manage presentation of a tubulovillous adenoma in the setting of a dual pancreas–kidney transplant. After resection, laparotomy, and extensive follow-up there have been no further recurrences of the tumor. Our knowledge of bladder tubulovillous adenomas in the setting of surgical modification and their clinical course has been furthered with the examination of this case.

## Abbreviation Used

CT: computed tomography
GFR: glomerular filtration rate
